# How Do Patients Who Fail First-Line TB Treatment but Who Are Not Placed on an MDR-TB Regimen Fare in South India?

**DOI:** 10.1371/journal.pone.0025698

**Published:** 2011-10-11

**Authors:** Sharath Burugina Nagaraja, Srinath Satyanarayana, Sarabjit Singh Chadha, Santosha Kalemane, Jyoti Jaju, Shanta Achanta, Kishore Reddy, Vishnu Potharaju, Srinivas Rao Motta Shamrao, Puneet Dewan, Zachariah Rony, Shailaja Tetali, Raghupathi Anchala, Nanda Kishore Kannuri, Anthony David Harries, Sachdeva Kuldeep Singh

**Affiliations:** 1 Office of the WHO Representative in India, New Delhi, India; 2 International Union Against Tuberculosis and Lung Diseases (The Union), Paris, France; 3 Impact Health Solutions, Hyderabad, India; 4 State Tuberculosis Training and Demonstration Centre, Hyderabad, Andhra Pradesh, India; 5 State Tuberculosis Cell, Directorate of Health Services, Hyderabad, Andhra Pradesh, India; 6 Medecins sans Frontieres, Medical Department (Operational Research), Brussels Operational Center, Luxembourg, Luxembourg; 7 Public Health Foundation of India (Indian Institute of Public Health- Hyderabad), Hyderabad, Andhra Pradesh, India; 8 Central TB Division, Ministry of Health and Family Welfare, Government of India, New Delhi, India; 9 Department of Infectious and Tropical Diseases, London School of Hygiene and Tropical Medicine, London, United Kingdom; McGill University, Canada

## Abstract

**Setting:**

Seven districts in Andhra Pradesh, South India

**Objectives:**

To a) determine treatment outcomes of patients who fail first line anti-TB treatment and are not placed on an multi-drug resistant TB (MDR-TB) regimen, and b) relate the treatment outcomes to culture and drug susceptibility patterns (C&DST).

**Design:**

Retrospective cohort study using routine programme data and *Mycobacterium TB* Culture C&DST between July 2008 and December 2009.

**Results:**

There were 202 individuals given a re-treatment regimen and included in the study. Overall treatment outcomes were: 68 (34%) with treatment success, 84 (42%) failed, 36 (18%) died, 13 (6.5%) defaulted and 1 transferred out. Treatment success for category I and II failures was low at 37%. In those with positive cultures, 81 had pan-sensitive strains with 31 (38%) showing treatment success, while 61 had drug-resistance strains with 9 (15%) showing treatment success. In 58 patients with negative cultures, 28 (48%) showed treatment success.

**Conclusion:**

Treatment outcomes of patients who fail a first-line anti-TB treatment and who are not placed on an MDR-TB regimen are unacceptably poor. The worst outcomes are seen among category II failures and those with negative cultures or drug-resistance. There are important programmatic implications which need to be addressed.

## Introduction

In India, out of the 1.2 million ‘new’ cases of tuberculosis (TB) notified in 2009, 14,991(1.8%) were reported to have failed the first line ‘anti-TB treatment drug regimen. Similarly among 289,756 re-treatment TB cases , 11,265 (4%) failed the first-line re-treatment drug regimen. [1].

Multi-drug resistant TB, MDR-TB (resistance to two of the potent first line anti-TB drugs, Isoniazid and Rifampicin) is one of the important causes for failure on TB treatment. In order to identify such patients early and manage them with an appropriate drug regimen, sputum specimens of all ‘new’ TB patients who are sputum smear positive at 5 months or more after the initiation of treatment (defined as having failed their first-line anti-TB treatment regimen)-, should be sent for sputum Culture and Drug Susceptibility Testing (C&DST) in an Revised National Tuberculosis Programme (RTNCP) accredited laboratory and patients should be placed on a re-treatment regimen while waiting for their C&DST results. Similarly, for ‘Re-treatment TB’ cases, sputum samples of patients who are smear positive at 4 months or more after the initiation of treatment (defined as having failed their first line anti-TB treatment) should be sent for C&DST and patients should be continued on a re-treatment regimen while awaiting the laboratory results.

As per RNTCP guidelines, patients with DST results showing resistance to rifampicin are considered eligible for MDR-TB treatment, irrespective of resistance to isoniazid, streptomycin, or ethambutol. In the absence of rifampicin resistance, patients simply continue their re-treatment regimen to completion [2], [3].

Of the 340 isolates tested by C&DST approximately 60% of the isolates were found to be susceptible to rifampicin and were not eligible for MDR-TB treatment, and hence were continued on the ‘re-treatment’ regimen. There is very little published information in India on how such patient's fare, as all of them are already showing signs of poor response to conventional first-line anti-TB treatment, and some of them have considerable non-rifampicin mono or poly-drug resistance. Such information is essential to guide the choice of continuing (or not) the current re-treatment regimen for such patients.

We thus conducted a retrospective cohort study in Andhra Pradesh, South India, to a) determine the treatment outcomes of patients who fail a first line anti-TB treatment regimen and are not placed on an MDR-TB regimen and b) relate their treatment outcomes to culture and drug susceptibility patterns.

## Methods

### Study setting

The study was conducted in seven districts (with a combined population of 18.4 million) in the state of Andhra Pradesh, South India, which are implementing RNTCP MDR-TB treatment services. The MDR-TB treatment services have been implemented in four of these seven districts since mid 2007 and the other 3 districts since early 2008.

### Study population, sampling

We selected all patients from these 7 districts who as per the programme guidelines were assessed as having drug resistance by C&DST at the two RNTCP accredited laboratories and were found not eligible for treatment with an MDR-TB treatment regimen. Patients registered during the period July 2008 to December 2009 were included in the study.

**Management of Tuberculosis patients who are not responding to TB treatment** RNTCP uses World Health Organization (WHO) recommended disease classification and treatment management guidelines. Patient management is guided by type of disease, sputum smear status and history of previous TB treatment as recommended by WHO. **Table 1** shows the categories of TB treatment regimens used for the treatment of TB under RNTCP. Reporting of TB treatment outcomes is done in a standardized manner (**Table 2**). All patients are treated under the supervision of a Direct Observation Treatment (DOT) provider. All doses during intensive phase are supervised, whereas during the continuation phase, only the first dose of the week is supervised while the remaining two doses are self administered by the patients themselves. Treatment adherence is assessed by verifying blister packs. While on treatment, patients undergo follow-up sputum examination at the end of intensive phase, two months into the continuation phase and at the end of treatment to assess the response to drug therapy. Any new patient who is sputum smear positive at five months or more after the initiation of therapy (initial treatment failures) and any re-treatment TB patient who is sputum smear positive four months or more after the initiation of therapy (re-treatment failures) are assessed for the presence drug resistance in an RNTCP accredited C&DST laboratory.

**Table 1:** Treatment regimens and times of follow-up sputum smear examinations in the Indian Revised National Tuberculosis Control Programme.

Category of Treatment	Type of patient	Treatment regimens***		Follow-up sputum examination
		Intensive Phase	Continuation phase	
Category I	New sputum smear positive	2(H_3_R_3_Z_3_E_3_)	4(H_3_R_3_)	2^nd^,4^th^ and 6^th^ month
	New seriously ill sputum smear-negative*			
	New seriously ill extra –pulmonary*			
Category II	Sputum smear positive Relapse	2(H_3_R_3_Z_3_E_3_S_3_)+1(H_3_R_3_Z_3_E_3_)	5(H_3_R_3_E_3_)	3^rd^,5^th^ and 8^th^ month
	Sputum smear positive failure			
	Sputum smear positive Treatment after default			
	Others			
Category III	New sputum smear-negative not seriously ill**	2(H_3_R_3_Z_3_)	4(H_3_R_3_)	2^nd^ month and 6^th^ month
	New sputum extra pulmonary not seriously ill**			

*In children, seriously ill sputum smear-negative Pulmonary Tuberculosis (PTB) includes all forms of sputum smear-negative PTB other than primary complex. Seriously ill extrapulmonary tuberculosis (EPTB) includes TB meningitis (TBM), disseminated TB, TB pericarditis, TB peritonitis and intestinal TB, bilateral extensive pleurisy, spinal TB with or without neurological complications, genitourinary TB, and bone and joint TB.

**Not seriously ill sputum smear-negative PTB includes primary complex. Not seriously ill EP-TB includes lymph node TB and unilateral pleural effusion.

***Prefix indicates month and subscript indicates thrice weekly.

**Table 2:** Definitions of treatment outcome.

**Cured:** Initially sputum smear-positive patient who has completed treatment and had negative sputum smears, on two occasions, one of which was at the end of treatment.
**Treatment completed**: Sputum smear-positive patient who has completed treatment, with negative smears at the end of the intensive phase but none at the end of treatment. Or: Sputum smear-negative TB patient who has received a full course of treatment and has not become smear-positive during or at the end of treatment.
Or: Extra-pulmonary TB patient who has received a full course of treatment and has not become smear-positive during or at the end of treatment.
**Treatment success**: includes cured and treatment completed together.
**Death:** Patient who died during the course of treatment regardless of cause
**Failure:** Any TB patient who is smear positive at 5 months or more after starting treatment. Failure also includes a patient who was treated with Category III regimen but who becomes smear positive during treatment.
**Defaulted:** A patient who has not taken anti-TB drugs for 2 months or more consecutively after starting treatment.
**Transferred out:** A patient who has been transferred to another Tuberculosis Unit/District and his/her treatment outcome is not known.

### Culture and Drug Susceptibility testing

The Intermediate reference laboratory at Hyderabad (Government sector) and Blue Peter research centre laboratory, Hyderabad were the culture and drug susceptibility testing (DST) laboratories that performed the DST. All the samples were collected by trained staff and transported to the laboratories in 1% cetylpyridium chloride (CPC) solution. These laboratories maintain a register to document the receipt of sputum specimens and the results of the C&DST.

These laboratories were accredited by RNTCP. Accreditation involves a pre-accreditation visit by a team of experts from the Tuberculosis Research Center (TRC), Chennai (a WHO Supra-National reference laboratory) which looks at the adherence of the laboratory to standard operating procedures laid down by the programme. The identification of *Mycobacterium tuberculosis* strains is based on growth rate, morphology, and susceptibility to para-nitrobenzoic acid. For drug susceptibility, the proportion method on Lowenstein-Jensen (LJ) is used for standard anti-tuberculosis drugs (isoniazid, streptomycin, rifampicin and ethambutol) and tested using standard procedures. For quality assurance, the standard *Mycobacterium tuberculosis* H37Rv strain was used. Both the laboratories had passed proficiency testing which involves re-testing and panel testing. The performance of both the laboratories was satisfactory as determined by standard concordance of >95% for Isoniazid and rifampicin and >90% for streptomycin and ethambutol. The laboratories also participated in the periodic proficiency testing programmes being conducted by a supra national laboratory (Tuberculosis Research Centre, Chennai).

### Data collection and analysis

We reviewed a) the laboratory registers at the C&DST laboratories which are the reference laboratories, b) the culture & DST register at district level which indicates the referrals made, and c) and the TB treatment cards and TB registers at sub-district level in which are documented the treatment outcomes. A line- list of all patients who had their C&DST done during the study period and whose DST results showed no resistance to rifampicin was prepared after ensuring the correctness of the records. Patients with negative C&DST results or no-growth on C&DST were also included in this data base. We collected baseline demographic data, the C&DST results and treatment outcomes of these line listed patients. The treatment outcomes that are reported for all cases in this study are a result of treatment with ‘retreatment regimen’. The data were cross verified by two investigators and compared for consistency; once it was found to be correct the data were entered in the Microsoft excel 2003. All variables were described by proportions and differences between independent groups were compared using Chi-square test and Fisher exact test as applicable by Epi-info software. Treatment outcomes were grouped as successful and adverse (death, failure, default and transferred out) and patients in each of these groups were compared. A ‘p value’ less than 0.05 were taken as statistically significant.

### Ethics approval

The study was approved by the ethics committee of the Public Health Foundation of India and the Ethics Advisory Group of The International Union Against Tuberculosis and Lung Disease, Paris, France. The study was determined to be a retrospective audit of the programme surveillance data in its records and reports, and permission was obtained from the programme managers at the state and national levels to access these data. Individual patient consent was deemed un-necessary by both the ethics committees. Electronic databases created for this analysis were stripped of personal health identifiers and maintained securely.

## Results

### Characteristics of the study population

There were 204 patients who fulfilled the study eligibility criteria of whom 2 patients could not be identified in the tuberculosis register and were subsequently excluded from the analysis. Of the remaining 202 patients, 58 failed a Category I treatment regimen, 139 patients failed a Category II treatment regimen and 5 patients failed a Category III treatment regimen. The median age of the patients was 35 years (range 9–70 years) and there were 143 (70%) males. All patients were treated with the recommended RNTCP re-treatment regimen.

### Treatment outcomes of the study population

Treatment outcomes as a result of initiating/continuing all patients on a re-treatment regimen are shown in Table 3. Among the 202 patients, the overall treatment success rate (cured or treatment completed) was 34%. The majority of the patients either failed the treatment again (42%) or died (18%). The treatment success rate of patients who failed a Category II treatment regimen (27%) was worse than patients who failed a Category I (47%) or Category III treatment (60%). Among the 15 patients who were below the age of 18 years, the treatment success rate was only 27% and majority of those with an adverse outcomes failed treatment (60%).

**Table 3:** Treatment outcomes of TB patients who failed a first line anti-TB treatment and were not placed on MDR-TB treatment regimens, Andhra Pradesh, India (July 08 to December 09).

Category of treatment	Treatment success	Failure	Died	Default	Transfer Out	Total No.	*p value*
	No. (%)	No. (%)	No. (%)	No. (%)	No. (%)		
Category I Failures	27 (47)	20 (34)	7 (12)	4 (7)	0 (0)	**58**	**Reference** $
Category II Failures	38 (27)	62 (45)	29 (21)	9 (6)	1 (1)	**139**	**0.008** *
Category III Failures	3 (60)	2 (40)	0 (0)	0 (0)	0 (0)	**5**	**0.45** **
**Total**	**68 (34)**	**84 (42)**	**36 (18)**	**13 (6)**	**1 (0)**	**202**	

*Chi-square test,

**Fisher exact test.

$ (In order to calculate ‘*p value*’ treatment success is compared with other outcomes).

### Relationship between treatment outcomes and C&DST patterns

Results of treatment outcomes in relation to C&DST are shown in Table 4. Of patients who failed a Category I and II treatment regimen (n = 197), 140 (71%) were culture positive with a DST pattern and 57 (29%) were culture negative. Among the 140 culture positive patients with a DST pattern, the treatment success rate was considerably worse among the patients with any drug resistance pattern (15%) compared with patients who had pan-sensitive organisms (37%). High failure rates were found among patients with any drug resistance (47%) compared with those who had pan-sensitive organisms (41%).

**Table 4:** Treatment outcomes and drug susceptibility patterns for first line anti-TB drugs amongst Patients who failed Category I and Category II anti-TB treatment and who did not receive an MDR-TB treatment regimen in Andhra Pradesh, India (July 08 to December 09).

Sensitivity Pattern	Treatment Success	Failure	Died	Default	Transfer out	Total	*p value*
	No. (%)	No. (%)	No. (%)	No. (%)	No. (%)	No.	
Pansensitive	29 (37)	32 (41)	11 (14)	6 (8)	1 (1)	79	Reference$
Any resistance	9 (15)	28 (47)	17 (29)	5 (8)	0 (0)	59	0.005#
Resistant to S* only	3 (33)	3 (33)	3 (33)	0 (0)	0 (0)	9	1.0§
Resistant to H* only	3 (15)	9 (45)	6 (30)	2 (10)	0 (0)	20	0.06#
Resistant to H and S	0 (0)	9 (64)	4 (29)	1 (7)	0 (0)	14	0.004§
Resistant to H and E*	0 (0)	2 (50)	0 (0)	2 (50)	0 (0)	4	0.29§
Resistant to S and E	1 (25)	2 (50)	1 (25)	0 (0)	0 (0)	4	1.0§
Resistant to SHE	2 (25)	3 (37.5)	3 (37.5)	0 (0)	0 (0)	8	0.7§
Negative	27 (47)	21 (37)	7 (12)	2 (4)	0 (0)	57	0.21#
NTM*	0 (0)	1 (50)	1 (50)	0 (0)	0 (0)	2	0.53§

*S-Streptomycin, H-Isoniazid, E-Ethambutol, NTM-Non tuberculosis mycobacterium;

# Chi square test;

§ Fisher exact test.

$ (In order to calculate ‘*p value*’ treatment success is compared with other outcomes.

Of the 5 patients who failed a Category III treatment regimen, 2 had a pan-sensitive organism, 1 had a strain with streptomycin mono-resistance, 1 a strain with isoniazid mono-resistance and 1 had negative C&DST pattern (not included in table 4). All patients (n = 3) with negative or pan-sensitive C&DST patterns had successful treatment outcomes.

## Discussion

This study shows that overall treatment outcomes of patients who fail a first line anti-TB treatment and who are not placed on an MDR-TB regimen are unacceptably poor. The worst outcomes were seen among category I and II failures placed on a re-treatment regimen. The findings have the following programmatic implications.

First, only one in three patients had treatment success on the current RNTCP re-treatment regimen. Thus, irrespective of drug sensitivity patterns, patients who fail on first line treatment in India have poor treatment outcomes if continued on the ‘re-treatment regimen’. Similar studies have been documented from other parts of the world [4] [5].

Second, under ideal conditions, failure in a well-run NTP should be infrequent in the absence of MDR-TB. The occurrence of failure is generally linked to programme factors such as poor adherence to treatment or poor drug quality [6]. We do not think that drug quality is a problem as RNTCP has well defined guidelines and drug procurement follows World Health Organization (WHO) pre-qualification criteria [7]. Although, we do not believe that adherence to treatment is a core problem, it may be necessary to consider measures, such as intensive supervision by supervisory staff which includes random blister pack checks of at least 50% of registered TB patients in a cohort every quarter until the outcome is declared, regular community driven patient provider meetings, periodic counseling for patients by counselors, timely incentives to DOT providers, introducing directly observed treatment even during continuation phase and treatment adherence review by local community leaders, as these may have an influence on the poor outcomes. The health care delivery systems that are responsible for the treatment services should be made more accountable for TB treatment outcomes.

Third, current RNTCP treatment guidelines stipulate that patients with failure are continued or are retreated with the ‘re-treatment regimen’. The programme must urgently re-evaluate this treatment strategy especially in patients without pan-sensitive organisms or in whom there are negative cultures. There is limited information in the published literature on how best to manage such patients. A possible option would be to screen such patients at more frequent intervals (for example, every month) using rapid diagnostic technology to ascertain if they have developed rifampicin resistance or not. If they do develop rifampicin resistance, they could then be changed to the MDR-TB treatment regimen. However, this proposed strategy has serious feasibility challenges. The recent introduction of GeneXpert into the market which allows detection of Rifampicin drug resistance within two hours is promising but is unlikely to be available at wide level for some time to come [8]. An alternative and rather immediate option would be to consider shifting all patients without pan-sensitive organisms and negative cultures to an MDR-TB treatment regimen upfront. This seems a logical option if the TB Programme is to give such patients the best possible chance of “treatment success”. This is also in line with current WHO guidelines about how to manage this problem [6].

Finally, the entire approach of using the category II re-treatment regimen, in our opinion, merits urgent review. According to current practice, the retreatment regimen involves adding one new drug (streptomycin) to an already failing regimen. This contradicts the basic principle that at least four drugs to which the TB bacilli are sensitive are needed to ensure effective cure and prevent the development of drug resistance [6], [9]. The current re-treatment regimen would seem in these circumstances to be at high risk of constituting dual or mono-therapy and as such is likely to amplify background or developing drug resistance. If this drug resistance pressure involves rifampicin and isoniazid, the practice may create new MDR-TB cases. The individual and public health implications including the further transmission of MDR-TB to households, health staff and the community are serious.

The strengths of this study are as follows: large numbers of district records were carefully reviewed and outcomes verified using patient cards; the findings come from the programme setting and are thus likely to reflect the operational reality on the ground; C&DST results were quality controlled; and we adhered to STROBE guidelines on reporting [10]. The limitations of the study are that C&DST patterns were not available for a proportion of patients and these patients were thus labeled DST negative. This reflects the current difficulties of “culture yield” related to current DST techniques. We also do not know why patients with pan-sensitive organisms had relatively poor outcomes, and this observation merits further investigation. Although our cohort also involves seven districts in Andhra Pradesh, the cohort is relatively small and these findings merit further validation using larger programme data-sets including other states in India. This would validate these findings and inform national policy in a robust manner.

In conclusion, overall treatment success rates in failure patients placed on a re-treatment drug regimen are poor and these results need to be urgently reviewed. One of the immediate logical options particularly for patients who fail re-treatment category II regimens would be to start on an empiric MDR-TB regimen until C&DST results become available.
